# Fabrication of Non-phospholipid Liposomal Nanocarrier for Sustained-Release of the Fungicide Cymoxanil

**DOI:** 10.3389/fmolb.2021.627817

**Published:** 2021-03-30

**Authors:** Zheng Zhang, Jun Yang, Qing Yang, Guangyong Tian, Zhong-Kai Cui

**Affiliations:** ^1^State Key Laboratory for Biology of Plant Diseases and Insect Pests, School of Bioengineering, Dalian University of Technology, Dalian, China; ^2^Institute of Plant Protection and Shenzhen Agricultural Genome Research Institute, Chinese Academy of Agricultural Sciences, Beijing, China; ^3^Guangdong Provincial Key Laboratory of Bone and Joint Degeneration Diseases, Southern Medical University, Guangzhou, China; ^4^Department of Otorhinolaryngology, The Third Affiliated Hospital, Southern Medical University, Guangzhou, China; ^5^Department of Cell Biology, School of Basic Medical Sciences, Southern Medical University, Guangzhou, China

**Keywords:** non-phospholipid liposomes, fungicide, sustained release, nanotechnology applications, cymoxanil

## Abstract

Liposome nanocarriers can be used to solve problems of pesticide instability, rapid degradation and a short period of efficacy. Cymoxanil with antifungal activity requires an ideal drug loading system due to its degradation issues. In this paper, cholesterol and stearylamine were used to prepare non-phospholipid liposomes (sterosomes) as a pesticide nanocarrier, and were characterized with field emission scanning electron microscopy (FE-SEM), X-ray powder diffraction (XRD), Fourier-transform infrared (FT-IR) spectrometer, size distribution, and ζ-potential. The results showed sterosomes were successfully loaded with cymoxanil. The loading efficiency and the drug-to-lipid ratio were 92.6% and 0.0761, respectively. Prolonged drug release was obtained for 3 days, improving the short duration of the drug itself. The addition of cymoxanil-loaded sterosomes in culture medium effectively inhibited the growth of yeast cells, which serve as model fungal targets. Sterosomes as nanocarriers significantly improved the stability and efficacy of cymoxanil, thus introducing practical and economically desirable strategies for the preparation of novel pesticide formulations.

## Introduction

Cymoxanil (CYM), 2-cyano-N-[(ethylamino)carbonyl]C-2-(methoxyiminoacetamide, is an efficient systemic fungicide against devastating plant diseases such as cucumber downy mildew (Ziogas and Davidse, 1987), and has been successful against Peronosporales resistance (Fayette et al., 2012). Cymoxanil is a cyanoacetamide foliar fungicide used extensively in combination with other fungicides in grape, tomato, pepper, lettuce, and so on. It is usually used to control downy mildew, late blight, and frost disease in crops (Huang et al., 2019). This kind of fungal disease is very difficult to prevent. The main reason is that these pathogens grow and reproduce very fast, then form a huge pathogen population in a short time, thus causing devastating damage. The strong sporangia can also spread the disease in a long distance through rain, airflow, and agricultural operation, resulting in unexpected disease (Kamoun et al., 2015). Cymoxanil exhibits advantages of high efficiency, low toxicity, and weak persistency, achieving penetrant and curative activity on target plants. However, the instability posts a challenge for an optimized carrier to realize its efficacy.

The primary mode of action of CYM, which shows a strong inhibitory effect on the growth of mycelium, still remains unknown (Klopping and Delp, 1980). Despite a long and intensive history of application, the sensitivity toward pH and temperature of CYM limits practical applications (Morrica et al., 2004). The half-life of CYM at 25°C is estimated to range between 0.02 days (pH = 9.0) and 148 days (pH = 5.0), indicating that CYM is only stable in acidic solutions (Roberts and Hutson, 1999). In addition, biodegradation and photochemical reaction further shorten its effective duration, significantly limiting application in agriculture fields (Cavalier et al., 1991).

To date, CYM, as a highly effective therapeutic agent, is compounded with other protective agents to enlarge the scope of prevention and delay the development of drug resistance because of the diversity of pharmacological behaviors (Samoucha, 1988; Samoucha et al., 1988; Evenhuis et al., 1996). However, the high sensitivity and rapid degradability of CYM are still unsolved, and result in rapid loss of efficacy in the field. Improvement of the bioactive duration of CYM, as well as reduction of pesticide pollution, have always been important issues in the field of agriculture and environmental chemistry. There is a need to develop new formulations for achieving efficient loading, protection and sustained release of drugs, to thus provide an effective strategy to solve problems such as drug loss and environmental pollution.

The application of nanotechnology in agriculture has become a popular research topic. Nanocarrier materials can help to sustain and control the release of pesticides better than traditional pesticide formulations, improving drug dispersion, efficacy and stability (Huang et al., 2018). Liposomes are perhaps the most well-studied nanocarriers for drug loading because they are easy to fabricate, monodisperse, and exhibit good biocompatibility (Xing and Zhao, 2018). However, conventional liposomes prepared with phospholipids are not conducive to agricultural applications in complex environments due to their limited stability and tendency toward oxidation and hydrolysis (Grit and Crommelin, 1993). It has been shown that mixtures of single-chain amphiphiles and high content sterols can form liquid-ordered (lo) lamellar phases (Cui and Lafleur, 2014). These bilayers are able to generate large unilamellar non-phospholipid liposomes which are nanoscale and possess a high specific surface area and exhibit good stability in the aqueous phase (Linsley et al., 2018). These novel sterosomes hold great promise as potential drug carriers in the agriculture sector.

In this study, novel sterosomes prepared from cholesterol (Chol) and stearylamine (SA) were employed to load CYM. The resulting CYM-loaded sterosomes were characterized for the morphology, loading efficiency, stability, dispersion and drug release performance. Release behavior analysis and bioassay experiments *in vitro* were carried out to evaluate the properties of sustained release and prolonged effective duration of CYM. The application of nanomaterials and related technologies provide an alternative to traditional agricultural products. Pursuing pesticide carriers that can promote the effective use of drugs is crucial to the development of new pesticide formulation technologies.

## Materials and Methods

### Chemicals

Cymoxanil was purchased from Heowns, Tianjin, China. As a commercial product, CYM/chlorothalonil dispersible concentrate (DC) was obtained from Jiadeli Science and Technology Company, Sichuan, China. Cholesterol (Chol), ethylenediaminetetraacetic acid (EDTA), tris(hydroxymethyl)aminomethane (TRIS), 2-morpholinoethanesulfonic acid monohydrate (MES) and dialysis membrane (3.5 kDa) were purchased from Solarbio, Beijing, China. Stearylamine (SA) and benzene (analytical grade) were supplied by Macklin, Shanghai, China. Methanol (analytical grade) and sodium chloride (NaCl) were purchased from Kermel, Tianjin, China. Yeast extract peptone dextrose (YPD) medium was used to culture yeast cells. Yeast extract was purchased from Oxoid, Hants, United Kingdom. Peptone was obtained from Aobox, Beijing, China. Dextrose was purchased from Kermel, Tianjin, China. Dimethyl sulfoxide (DMSO) and acetonitrile (HPLC grade) were purchased from Sigma-Aldrich, Saint Louis, MO, United States. Deionized water was obtained from a Milli-Q water system (Master Q15, Hitech Instruments CO., Shanghai, China) and was used for all reactions and treatment processes.

### Preparation of Sterosomes

Sterosomes were prepared as previously described (Cui et al., 2017). Briefly, dissolve SA and Chol (1/1 molar ratio) in benzene/methanol 90/10 (v/v). Freeze the solution in liquid nitrogen and lyophilize for at least 16 h. Hydrate the freeze-dried powder mixtures with a MES/TRIS buffer (TRIS 50 mM, MES 50 mM, NaCl 130 mM, EDTA 0.5 mM, pH = 5.0). Cycle temperature five times from liquid nitrogen (LN) temperature to ∼70°C (about 1 min in LN and heat for 10 min), and vortex (∼30 s) between successive cycles. After hydration, measure and readjust the pH to 5.0. Sonicate the hydrated suspensions at 40% intensity and an 80% duty circle for 20 min. The final concentration of sterosomes suspensions was 20 mg/mL. CYM-loaded sterosomes were prepared by adding CYM (dissolved in DMSO) to sterosome suspensions, and stirring overnight at room temperature.

### Encapsulation and Loading Properties

The concentration of CYM was detected by absorbance (240 nm) with HPLC (Agilent 1200, Beijing, China). The details of the method are listed in Table 1 (Morrica et al., 2004). An equal volume of acetonitrile was added to CYM-loaded sterosome suspensions and shaken for 10 min to lyse the sterosomes. The supernatant was passed through a 220-nm filter and the flow-through was injected into the HPLC. The amount of CYM in the drug-loaded sterosomes was detected and calculated by comparing to a standard curve. The loading efficiency (LE) was defined as the ratio of actual versus theoretical amount of CYM in the drug-loaded sterosomes, as described by Equation 1. The drug to lipid ratio (D/L) was defined as the ratio of actual amount of CYM versus total amount of lipids, as described by Equation 2.

**TABLE 1:** HPLC methods used for measuring cymoxanil (CYM) concentrations.

**Factors**	**Parameter**
Column	Hypersil BDS C18 250 × 4.6 mm, 5 μm
Flow rate	1.0 mL/min
Detection	240 nm, UV
Injection volume	15 μL
Isocratic	Acetonitrile/Water (30/70 v/v)

(1) $$\text{L}\,E=\frac{\mathrm{Actual}\,\mathrm{Amount}\,\mathrm{of}\,\mathrm{CYM}}{\mathrm{Theoretical}\,\mathrm{Amount}\,\mathrm{of}\,\mathrm{CYM}}=100\%$$

(2) $$D/L=\frac{\mathrm{Actual}\,\mathrm{Amount}\,\mathrm{of}\,\mathrm{CYM}}{\mathrm{Total}\,\mathrm{Amount}\,\mathrm{of}\,\mathrm{lipids}}$$

### Characterization of CYM-Loaded Sterosomes

Size and ζ-potential were measured with a ZS90 (Malvern Instruments, United Kingdom). The sample was prepared with 1 mL sterosomes and CYM-loaded sterosomes. The change of size and ζ-potential after CYM loading showed dispersion and stability properties.

Before morphology analysis, sterosomes and CYM-loaded sterosome suspensions were freeze-dried overnight to yield uniform powders. Field emission scanning electron microscope (FE-SEM) images were recorded on a NOVA NanoSEM 450 (FEI, United States) under operation at an acceleration voltage of 20 kV.

X-ray powder diffraction (XRD, SmartLab 9KW, Japan) confirmed the crystallinity of sterosomes and CYM-loaded sterosomes in a 2θ range of 10∼80° with Cu-Kα radiation of 1.54 Å, an operating voltage of 40 kV, and a current of 40 mA.

Fourier-transform infrared (FT-IR) spectroscopy was used to analyze the functional groups of sterosomes and CYM-loaded sterosomes with an FTIR Spectrometer (6700, Thermo Fisher Scientific, United States).

### Release Behaviors of CYM-Loaded Sterosomes

A concentration of 1 mg/mL CYM in the drug-loaded sterosomes was transferred into a dialysis membrane (3.5 kDa), which was placed in a beaker containing 200 mL MES/TRIS buffer (pH = 5.0), at room temperature. An aliquot of the sample (20 μL) was collected from the beaker at the pre-determined time points to measure the CYM concentration, and an equal volume of fresh MES/TRIS buffer was replenished. Free CYM solution served as a negative control group and CYM/chlorothalonil DC (D/L 0.0204) was used as a commercial positive control group, which is mainly applied for the control of cucumber downy mildew. Three independent experiments were carried out. The quantification was calculated with the following equation:

(3) $$E_{r}=C_{t}=V_{0}/M_{p}$$

where *E*_*r*_is the cumulative release of CYM, %; *C*_*t*_ is the concentration of CYM in the MES/TRIS buffer at time *t*, mg/mL; *V*_0_ is the volume in the membrane, mL; and *M*_*p*_ is the mass of CYM in the carrier, mg.

### Antifungal Activity Analysis

*Saccharomyces cerevisiae* (*S. cerevisiae*) served as a model fungus system to evaluate the sustained release of CYM-loaded sterosomes resulting from the statistics of cytotoxicity assay (Ribeiro et al., 2000; Fai and Grant, 2009). Each sample (CYM solution, CYM-loaded sterosomes, and CYM/chlorothalonil DC) was prepared and dispersed in YPD medium via dilutions at the effective testing concentrations of CYM, 50 mg/L (Ribeiro et al., 2000). The same concentration of sterosomes was added to evaluate the toxicity to yeast cells. The blank group was only cells in YPD medium, displaying the normal growth cycle of cells. Then *S. cerevisiae*grew at 30°C, 200 rpm, and the growth status of the cells was detected by OD_620_.

## Results and Discussion

### FE-SEM Imaging

**FIGURE 1:**
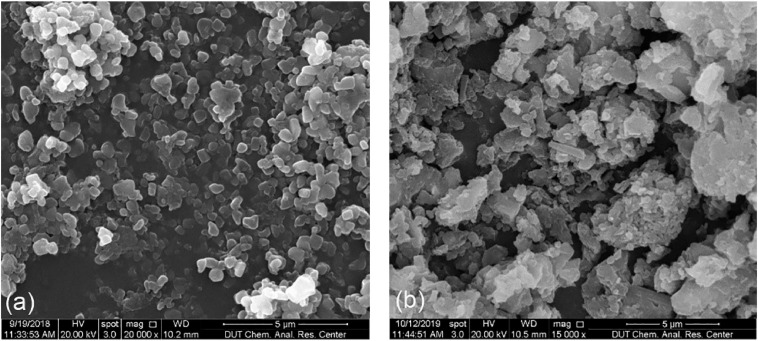
FE-SEM images of **(a)** sterosomes; **(b)** CYM-loaded sterosomes. Scale bar = 5 μm.

The morphology of prepared CYM-loaded sterosomes was characterized using FE-SEM (Figure 1). The well-distributed nanoparticles were observed after lyophilization. A few crystals from dried MES/TRIS buffer were present among sterosome powders. The size of sterosome particles fell under the nanometer scale, resulting in a high specific surface area, which provides more space for interactions between drugs and nano-carriers. After loading with CYM, the appearance of sterosomes became less regular, with the reduction of sphericity, indicating the successful loading of CYM, modifying the surface of sterosomes.

### Size and ζ-Potential Analysis

**FIGURE 2:**
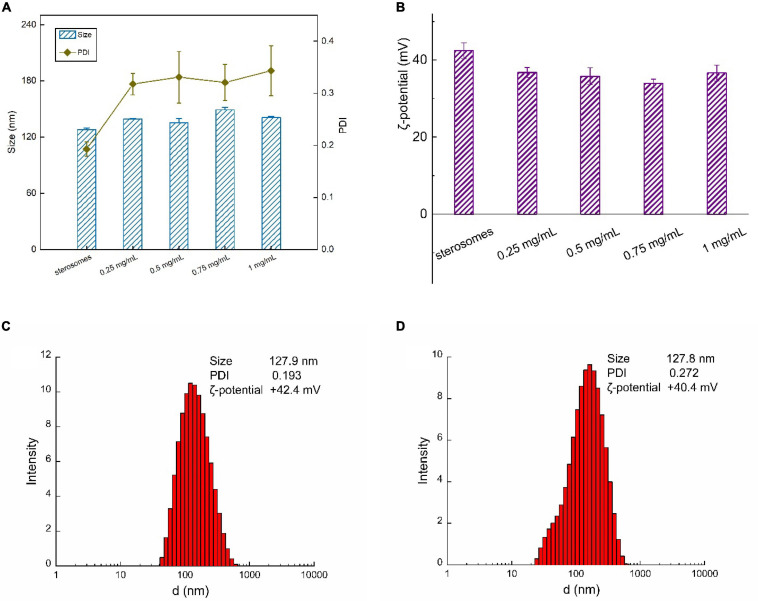
Size and ζ-potential of CYM-loaded sterosomes with various CYM concentrations: **(A)** size distribution and PDI; **(B)** ζ-potential with various CYM concentrations ranging from 0.25 to 1 mg/mL; size distribution and ζ-potential of **(C)** sterosomes; **(D)** CYM-loaded sterosomes (1 mg/mL CYM).

CYM-loaded sterosomes were prepared by adding CYM to sterosome suspensions. Various amounts of CYM were added to evaluate the effects on particle size distribution and ζ-potential properties. With the increase of the CYM concentrations over the range of 0.25∼1 mg/mL, the particle size of sterosomes exhibited no obvious change, while the polydispersity index increased after CYM loading (Figure 2A). Moreover, the ζ-potential of sterosomes after CYM loading decreased, indicating that some of the CYM was complexed on the surface of sterosomes (Figure 2B). The electrostatic interactions between the SA/CholSterosomes and the cymoxanil were the main driving force for complexation.

Dynamic light scattering (DLS) methods revealed more details of the distribution of sterosomes in suspensions. The nanoparticle size of sterosomes ranged between 120 and 130 nm (Figure 2C). One of the most unique properties of Chol in relation to the SA/Chol structure is its ability to induce the formation of a liquid-ordered (lo) lamellar phase (Carbajal et al., 2013). In the equimolar mixture at pH < 9, protonated SA is well mixed with Chol molecules, leading to the formation of stable lo lamellar phases (Cui and Lafleur, 2014). Then homogeneous sterosomes were obtained after ultrasonic dispersion. The ζ-potential for empty sterosomes was +42.4 mV, arising from the protonated amine groups of SA. A small decrease of ζ-potential for CYM-loaded sterosomes (+40.4 mV) was associated with the loading of CYM, covering the exposed protonated of SA (Figure 2D). According to the data of PDI, the dispersity of the sterosomes in buffer was relatively uniform because of the electrostatic repulsion among particles. CYM-loaded sterosomes are uniformly dispersed in the MES/TRIS buffer (pH = 5.0) and present good stability.

### XRD Analysis

**FIGURE 3:**
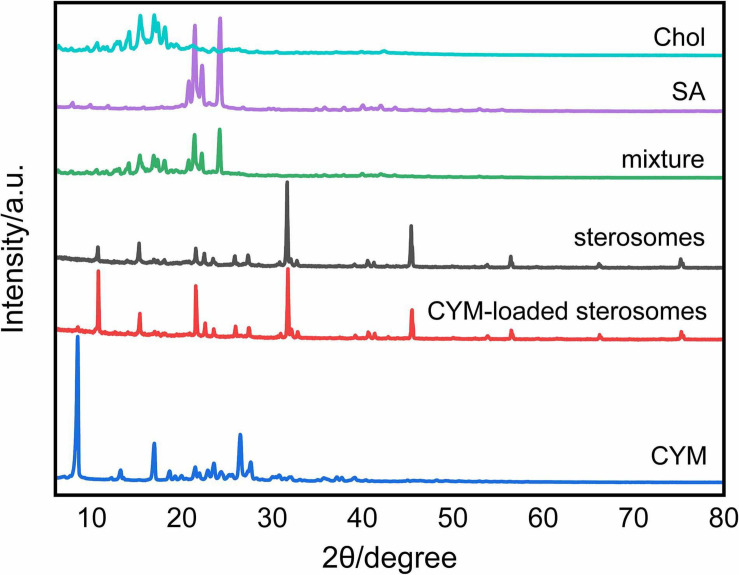
X-ray diffraction patterns of different components of sterosomes and CYM-loaded sterosomes.

The XRD patterns depict the crystalline structure of sterosomes to show self-assembly behaviors with SA and Chol. It is shown that SA and Chol presented different crystalline structures (Figure 3). The mixture showed overlapped diffraction patterns of SA and Chol, which proved that only physical mixing of SA and Chol took place without any intermolecular assembly arrangement. However, sterosomes presented a completely different crystalline structure, suggesting that successful self-assembly between SA and Chol occurred during hydration and a new sterosome structure was formed. After CYM loading, the crystalline structure of CYM-loaded sterosomes at 2θ values barely changed (Figure 3). The addition of CYM did not alter the structure and integrity of sterosomes.

### FT-IR Analysis

**FIGURE 4:**
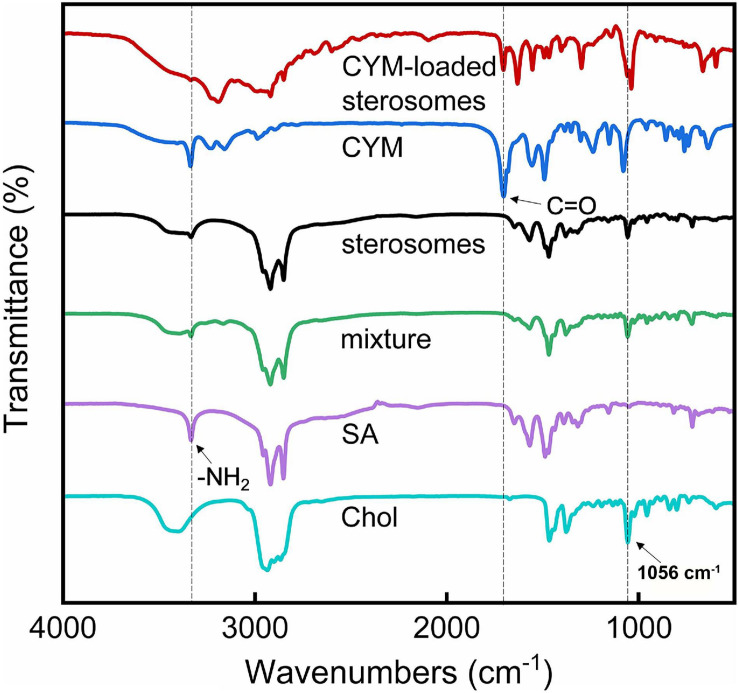
Fourier-transform infrared (FT-IR) spectroscopic spectra of different components of sterosomes and CYM-loaded sterosomes.

Fourier-transform infrared (FT-IR) spectroscopic analysis was carried out for further characterization of CYM-loaded sterosomes. FT-IR spectra of SA/Chol mixtures before and after hydration showed peaks of SA around 1,420∼1,480 cm^–1^ and 2,700∼3,000 cm^–1^ (Figure 4), indicating the structure of the aliphatic chain. The peaks in the area of 3,333 cm^–1^ showed the presence of an -NH_2_ group in SA. The peak at 1,056 cm^–1^is ascribed to the ring deformation of Chol. Compared with the mixture, sterosomes obtained by hydration showed the same peaks, which confirmed that the structural change caused by the self-assembly of SA and Chol belonged to the change of physical phase transition without the change of chemical functional groups. Furthermore, CYM showed a peak at 1,706 cm^–1^, associated with the ketone (C = O) groups, and CYM-loaded sterosomes also presented ketone (C = O) groups (Figure 4), indicating prominent cover of CYM on the sterosomes.

### CYM Loading Analysis

The CYM loading capacity of sterosomes is listed in Table 2. With the increased concentrations of CYM, the LE and D/L rose to 92.6% and 0.0761, respectively. The predominant interaction between drugs and sterosomes was surface electrostatic attraction. Typically, higher positive surface charge density leads to higher drug uptake efficiency and a more stable system (Cui et al., 2012). After CYM was added to sterosome dispersion, it was stirred overnight to facilitate its complete distribution in the buffer. Then, we measured the actual CYM content in the system. The results confirmed that CYM-loaded sterosomes with 1 mg/mL CYM achieved the highest LE and D/L among all of the experimental groups over the investigated concentration range, and a value much higher than that of the commercial product CYM/chlorothalonil DC (D/L 0.0204). Due to the limited solubility of CYM, ∼1 mg/mL, no higher concentration was accessible to reach a loading plateau.

**TABLE 2:** Loading parameters of CYM-loaded sterosomes.

	**Concentration of CYM (mg/mL)**			
	**0.25**	**0.5**	**0.75**	**1.0**
LE (%)	78.7 ± 4.3%	81.3 ± 6.3%	88.4 ± 2.2%	92.6 ± 2.6%
D/L	0.0162 ± 0.92 × 10^–3^	0.0336 ± 2.5 × 10^–3^	0.0549 ± 1.3 × 10^–3^	0.0761 ± 2.1 × 10^–3^

### Release Behaviors of CYM-Loaded Sterosomes

**FIGURE 5:**
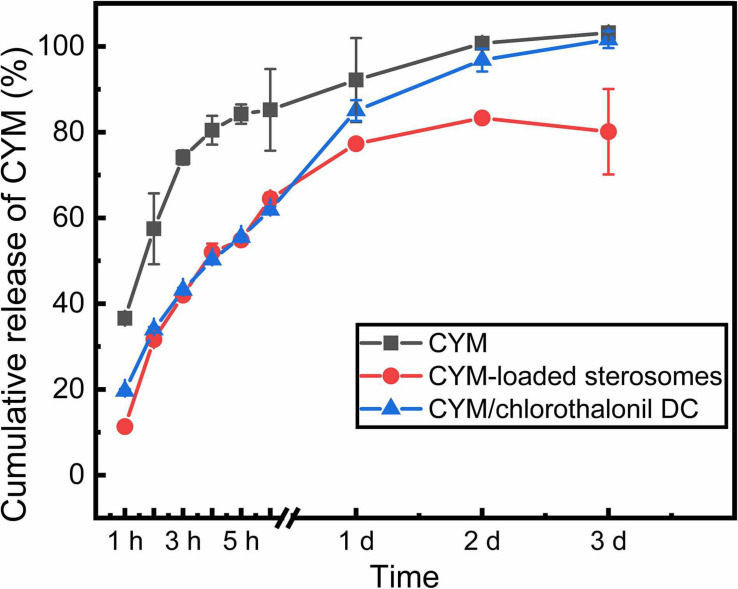
Release behaviors of CYM-loaded sterosomes (pH = 5.0) for 3 days compared with free CYM solution and CYM/chlorothalonil DC.

Comparison of the sustained release capacities of CYM-loaded sterosomes, free CYM solution and CYM/chlorothalonil DC at room temperature was performed (Figure 5). At the same concentration of CYM, the release rate of free CYM solution group was the fastest, on account of rapid partition under high osmolar pressure. In the first 6 h, both CYM-loaded sterosomes and CYM/chlorothalonil DC showed significant sustained release capacity. After 1 day, 80% of CYM was released from CYM/chlorothalonil DC and the loaded drug could be released completely within 3 days. However, the equilibrium was essentially reached in 24 h for CYM-loaded sterosomes and nearly 80% of the drug after 3 days was released. The surface interaction between CYM and sterosomes effectively reduced the drug release rate. The remainder of CYM remained on the surface of sterosomes, supposedly to improve its pharmacological action with carrier shift. Our newly developed CYM exhibited enhanced drug stability and better sustained release behavior compared to the commercially available product.

### Antifungal Activity Analysis

**FIGURE 6:**
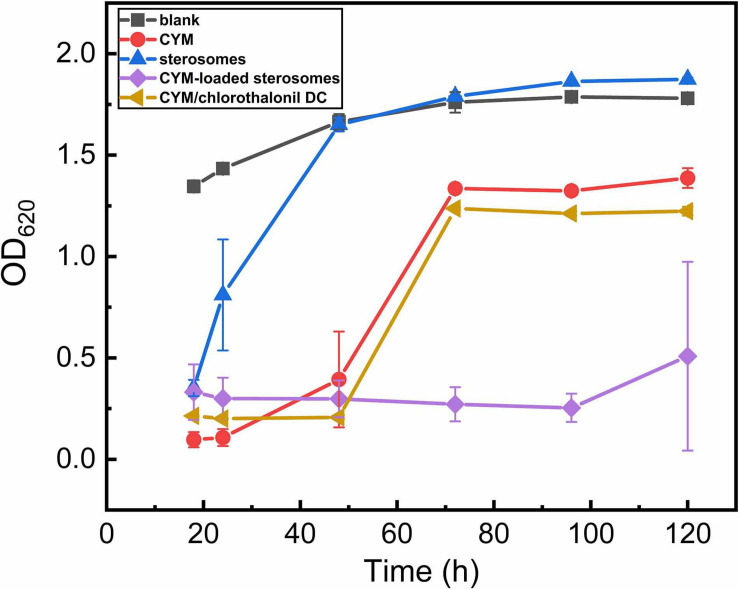
Effects of CYM-loaded sterosomes on growth parameters of the *S. cerevisiae* yeast in yeast extract peptone dextrose (YPD) medium.

CYM exhibited high efficacy against the yeast strain *S. cerevisiae* with an IC_50_ of 50 mg/L. Hence. in the pharmacological activity of CYM-loaded sterosome experiments, *S. cerevisiae*was used as the model fungus by detecting OD_620_ of culture medium to reflect the growth status of the cells (Figure 6). As the blank curve, after 48 h of normal yeast growth, the logarithmic growth stage ended, and it reached a plateau growth stage. However, affected by free CYM solution, the inhibition of yeast growth lasted for only 48 h, which confirmed that CYM was only effective for a relatively short duration. Then, after 72 h, the yeast growth reached a stable stage and remained constant. CYM/chlorothalonil DC showed the same inhibition effect as the CYM group. Surprisingly, the effective inhibition period of CYM-loaded sterosomes was extended to 120 h, thus significantly increasing the efficacious duration. Although the positive charge of sterosomes can facilitate intracellular uptake, it also associates with cytotoxicity resulting from the generation of reactive oxygen intermediates, toxic oxidative bursts, and a disruption of cellular and sub-cellular membrane functions (Campbell, 1983; Dokka et al., 2000; Romoren et al., 2004). It is supposed to have no significant effect on cell viability with sterosomes added (Cui et al., 2015). Interestingly, a certain inhibitory effect was observed with the pure sterosomes added during the early stage of yeast growth, resulting in the right shift of the yeast growth curve, and it was then consistent with the blank group after 48 h. After sterosomes were added, yeast cells aggregated in the culture medium, which could be caused by the electrostatic interactions between yeast cells and sterosomes. This is because of the negative charges on the surface of the cell membrane, which facilitates the complexation of cationic sterosomes to accumulate on the cell surface. This behavior is supposed to affect cell mass transfer and metabolism, thereby delaying yeast growth.

## Conclusion

In summary, this study focused on nanovehicles for loading small molecular pesticides. These non-phospholipid sterosomes were 120∼130 nm formulated with SA and Chol, which self-assembled in the MES-TRIS buffer with high dispersity. The LE and D/L reached 92.6% and 0.0761, respectively. The release rate was much slower than that of commercial CYM/chlorothalonil DC and reached nearly 80% after 3 days. The inhibition of CYM-loaded sterosomes on *S. cerevisiae* growth was extended to 120 h, which was 2.5 times more than that of free CYM solution. This research provides a new perspective to overcome the barriers of drug stability and drug efficacy. The application of sterosomes in the sustained release of various pesticides is beneficial to agricultural prevention and control.

## Data Availability Statement

The original contributions presented in the study are included in the article/supplementary material, further inquiries can be directed to the corresponding author/s.

## Author Contributions

JY, QY, and Z-KC: conceptualization, supervision, and project administration. JY, ZZ, and Z-KC: methodology. ZZ, JY, QY, GT, and Z-KC: validation, formal analysis, investigation, resources, original draft preparation, review and editing, and visualization. JY and ZZ: data curation. JY and Z-KC: funding acquisition. All authors contributed to the article and approved the submitted version.

## Conflict of Interest

The authors declare that the research was conducted in the absence of any commercial or financial relationships that could be construed as a potential conflict of interest.
